# Supplementary material for: Heterogeneous integration for on-chip quantum photonic circuits with single quantum dot devices
Source: Nat Commun. 2017 Oct 12;8:889. doi: 10.1038/s41467-017-00987-6 (PMC5715121; doi:10.1038/s41467-017-00987-6)
Supplement: Supplementary file 1 — Supplementary Information [file 41467_2017_987_MOESM1_ESM.pdf]

Supplementary Note 1 - Quantum photonic integrated circuits with quantum dots

The considerable potential of InAs/GaAs quantum dots (QDs), both for triggered single-photon generation and as quantum logic elements, has spurred the development of a number of platforms that seek to incorporate these QDs within photonic circuits. A direct method is to develop both active and passive components within the same material system. Along these lines, monolithic GaAs-based quantum photonic circuits with on-chip quantum dot-based single-photon sources have been demonstrated by a number of research groups [1]. Two general approaches have been adopted. In the first, passive circuits are composed of low-index-contrast GaAs/AlGaAs ridge waveguides produced on top of a GaAs/AlGaAs substrate [2, 3]. Such waveguide geometries can be produced with relatively straightforward fabrication processes, relying solely on epitaxial growth for vertical optical confinement and a single etching step for lateral confinement. Due to the small vertical refractive index contrast that is achievable through growth, waveguide cross-section dimensions are typically of the order of microns. While lower refractive indices are generally desirable for minimizing scattering losses in propagation, large mode field diameters (and large mode volumes in the case of cavities) translate into less compact devices, less effective light-matter interactions, and less effective geometrical control of waveguide dispersion (relevant for on-chip nonlinear optics). In particular, weak vertical confinement means more effective dipolar coupling to substrate radiative modes, translating into a reduced β-factor for radiating dipoles (see main text). Distributed-feedback reflector-based geometries such as in ref. 2 can ameliorate this, however the achievable mode-field diameters are also limited by the relatively small index contrast between GaAs and AlGaAs. All of these issues are to great extent circumvented in the second approach, in which circuits composed of suspended GaAs waveguides surrounded by air or vacuum, either of the channel [4] or photonic crystal [5] types (or both), are implemented. In this case, the strong index contrast allows strong transverse field confinement in waveguides of cross-sectional dimensions of the order of hundreds of nanometers. Small modal areas and cavity mode volumes can be achieved, meaning stronger light-matter interactions and higher β-factors for radiating dipoles [6], together with strong geometry-based control of waveguide dispersion. An important limitation of such an approach, however, is the losses due to scattering at the etched sidewalls, which can be considerably higher due to the strong index contrast between the semiconductor and the air. A second issue is that the fragility of suspended GaAs structures imposes limits on the dimensions of free-standing circuits, requiring support structures such as tethers or transitions to non-suspended waveguides, which may induce significant scattering losses [5]. Fabrication, device handling, and further integration with other types of on-chip elements are also more cumbersome in this case. An additional challenge common to both approaches is that passive circuits are produced in the same material layer that contains the QDs. Because there is no strict separation between active and passive portions of the photonic circuit, the population of QDs inside the passive section can contribute to excess optical absorption.

As discussed in the main text, our heterogeneous integration platform offers essentially all the advantages of the two approaches described above, while addressing many of the aforementioned challenges. The large refractive index contrast between GaAs and Si_3N_4 allows for strong modal confinement within the GaAs layer so that large β factors can be achieved in the active quantum dot region. This large refractive index contrast is, in addition, achieved without requiring devices to be undercut, improving the mechanical and thermal stability of the system, particularly as the number of integrated elements increases. Furthermore, complete removal of the GaAs material outside in the passive regions avoids excess optical absorption due to the background QD ensemble. Within the passive sections, the large refractive index contrast between Si_3N_4 and SiO_2 enables the dispersion engineering and large effective nonlinearity needed for nonlinear optics applications, such as frequency downconversion of the QD emission to the 1550 nm telecom band [7]. Such nonlinear optics applications can in principle be implemented in suspended GaAs photonic circuits, though the much wider bandgap of Si_3N_4 and SiO_2 in comparison to GaAs-based materials ensures that two-photon absorption, an important factor in nonlinear nanophotonic devices, is negligible over a wide range of wavelengths. Coupling off-chip to optical fibers can also be optimized, as our platform is compatible with end-fire approaches that utilize inverse tapers and symmetric low-index claddings [8, 9]. In particular, devices can be designed to admit a full SiO_2 cladding rather than the current top air cladding, with additional processing likely consisting of a single additional PECVD deposition step.

To make the distinction between the homogeneous GaAs-based photonic circuit platforms described above and our hybrid geometry more evident, in Supplementary Figures 1a-e we plot field profiles for representative waveguides of all types mentioned above. It is visually apparent that the modal areas achieved in our platform (Supplementary Figure 1d) are comparable to those obtained with suspended waveguides (Supplementary Figure 1a), of either channel [4] or photonic crystal [5] kind. The modal areas of our GaAs-on-Si_3N_4/SiO_2 waveguides are considerably smaller than the modal areas of all of the GaAs-based waveguides on GaAs/AlGaAs substrate [2, 3], shown in Supplementary Figures. 1b and c. More quantitatively, we display, for the plotted fundamental modes in each geometry, the effective area $A_{\text{eff}} = \int \epsilon_r |\mathbf{E}|^2 dS / \max\{|\mathbf{E}|^2\}$, where $\mathbf{E}$ is the electric field, ϵ_r is the relative permittivity and the integral is taken over the waveguide cross-section. This quantity is inversely proportional to the maximum interaction strength between the guided mode and a single quantum emitter inside the waveguide [11], and essentially serves as a measure

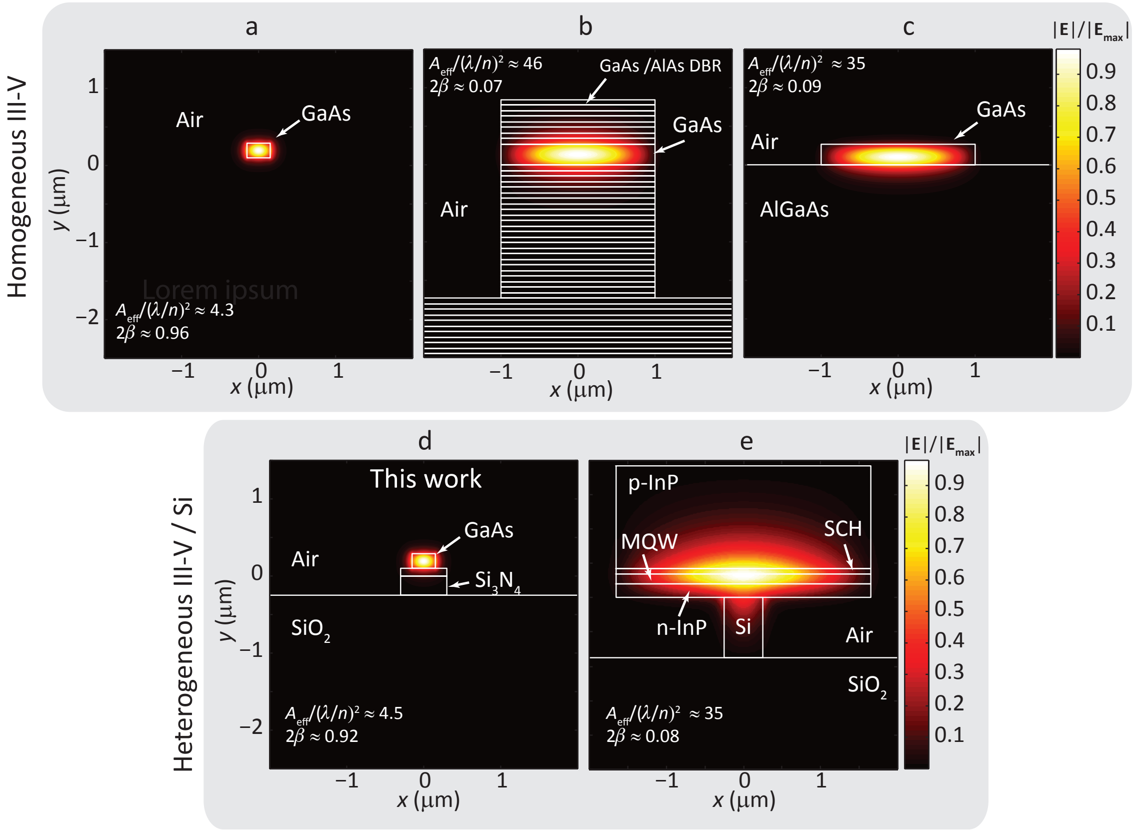

Supplementary Figure 1: **Comparison of waveguide geometries for single quantum dots**. Electric field profiles for the fundamental modes of **a**, suspended GaAs [4]; **b**, distributed feedback reflector (DBR) ridge [2]; **c**, GaAs/AlGaAs ridge [3]; **d**, GaAs / Si_3N_4 hybrid waveguides; and **e**, InP/InGaAsP/Si hybrid waveguides [10], with corresponding effective areas (normalized by $(\lambda/n)^2$, the corresponding squared wavelength in the semiconductor) and β-factors. Notes: 1) Photonic crystal waveguides [5], at frequencies away from slow-light band-edges, have similar cross-sectional modal profiles, effective areas and β-factors as the suspended channel guide in **a**. 2) The GaAs/AlGaAs ridge waveguide in **c** was chosen to have the same width as the DBR guide in **c**, for a fairer comparison; the original guide from ref. 3 had a width of 20 μm, which leads to a significantly larger effective area and lower β. 3) The InP/InGaAsP/Si hybrid geometry shown in **e** is typical for Si photonic laser geometries at telecom ($\approx$ 1550 nm) wavelengths. MQW: multi-quantum well. SCH: separate confinement heterostructure.

of the field concentration at the emitter location, assumed to be at the field maximum (an alternative definition that includes ϵ_r^2 in the denominator, analogous to that used for the effective volume in cavities, would yield effective areas smaller by a factor of $n^2 \approx (3.5)^2$, with n the refractive index of the III-V medium). We also provide the β-factor for each geometry. It is clear that modal areas in the suspended and hybrid geometries are roughly an order of magnitude smaller than in the GaAs geometries. In addition, $\beta > 90$ % is only achieved in suspended or hybrid structures. Both features are achieved through the high refractive index step between either air or Si_3N_4, which provides a sufficiently strong vertical confinement.

Lastly, while heterogeneous integration of active III-V layers and passive silicon-on-insulator waveguides has become an important technology in classical photonics [10, 12, 13], as noted in the main text, the specific geometries typically used are not necessarily suitable for quantum photonics. To illustrate this point, we make a comparison with geometries typical of Si photonic lasers [10] as shown in Supplementary Figure 1e. In this hybrid geometry, the Si ridge provides low-loss waveguiding, whereas the III-V layer provides optical gain through an InGaAsP multi-quantum well (MQW) heterostructure which is electrically pumped through the top and bottom p- and n-type InP layers. An InGaAsP separate confinement heterostructure (SCH) above the MQW acts as guiding core, providing a weak vertical optical confinement that results in the mode shown in the Figure, extending deeply into the p-InP layer. The p-InP layer is necessarily made thick in order to minimize propagation losses due to absorption at the electrical contact layers which top the III-V stack (not shown in the figure, generally consisting of a highly p-doped InP contact and a metal layer [12]). The effective area for such a mode is of the order of $35(\lambda/n)^2$, considerably larger than obtained with our strongly confining hybrid geometry, indicating a considerably smaller light-matter interaction strength. The

$2\beta \approx 0.08$ is also considerably lower. Both of these factor indicate that this geometry is far from optimal for probing single embedded emitters. It is worth pointing out that such large effective areas and low β-factors are comparable to those achieved in purely GaAs-based geometries with weak modal confinement discussed above.

While all of the fabrication steps that we have demonstrated in this work are scalable, the random nature of the in-plane spatial locations of self-assembled InAs/GaAs QDs is a limitation on the overall yield and ability to, for example, integrate multiple QD sites together within a passive Si_3N_4 circuit. Going forward, developments in site-controlled QD growth [14–16] will help to address this issue, and in general will be compatible with our fabrication approach. In the near-term, we can envision pre-characterization of QDs after creating the bonded wafer stack, such that GaAs devices are only created in regions for which desirable single QD behavior has been confirmed. In particular, photoluminescence imaging [17] has been confirmed as a technique for locating the position of single InAs/GaAs QDs with respect to alignment features, and recent implementations [18] have demonstrated the location of single QDs within $\approx 5000\ \mu m^2$ spatial regions with sub-10 nm positioning uncertainty, with typical image acquisition times of 1 s. We anticipate that such an approach can enable a higher throughput than pick-and-place techniques [19–22], which also require pre-screening of the quantum emitters along with the additional assembly steps.

We have summarized all of the differences between our platform and others in Supplementary Table 1.

	Low-loss propagation	Strong mode confinement and light-matter interaction	Mechanical Stability	Scalable top-down fabrication	Wideband optical nonlinearity
Suspended GaAs		×		×	see caption
DBR ridge			×	×	see caption
GaAs/AlGaAs ridge			×	×	see caption
Ref. 19	×		×		×
This work	×	×	×	×	×

Supplementary Table 1: **Comparison of existing platforms for integrated quantum photonics**. While III-V materials can have large $\chi^{(2)}$ or $\chi^{(3)}$, two-photon absorption (TPA) might limit the useful operating wavelength range of nonlinear optical phenomena. For GaAs, for example, pumps at wavelengths above 1700 nm are generally required to avoid TPA.

Supplementary Note 2 - Fabrication Details

To produce the starting wafer stack shown in Fig. 3a of the main text, we utilized the low-temperature plasma-activated direct wafer bonding procedure of ref. 12. The layer stack for the two wafers that are bonded in this procedure, one silicon-based and one GaAs-based, are given in Supplementary Tables 2 and 3 respectively. The Si_3N_4 layer of the silicon-based stack in Supplementary Table 2 was grown with low-pressure chemical vapor deposition, and the epilayer stack of Supplementary Table 3 was grown via molecular beam epitaxy.

Layer	Material	Thickness (nm)
Waveguide	Si_3N_4	550
Bottom cladding	Thermal SiO_2	3000
Substrate	Si	-

Supplementary Table 2: **Si_3N_4 wafer stack**

Layer	Material	Thickness (nm)
Surface cap	GaAs	10
Waveguide top	$Al_{0.30}Ga_{0.70}As$	40
Waveguide top	GaAs	74
Quantum well	$In_{0.15}Ga_{0.85}As$	6
Quantum dot	InAs	2.4 monolayer
Barrier	$In_{0.15}Ga_{0.85}As$	1
Waveguide bottom	GaAs	74
Sacrificial layer	$Al_{0.30}Ga_{0.70}As$	50
Sacrificial layer	$Al_{0.70}Ga_{0.30}As$	1500
Substrate	GaAs	-

Supplementary Table 3: **GaAs Epilayer Stack**

A $\approx$ 30 nm layer of SiN was deposited on top of cleaved ($\approx$ 5 mm^2) pieces of the III-V epiwafer with plasma-enhanced chemical vapor deposition (PECVD). Contact lithography followed by reactive ion etching in a $CHF_3/O_2/Ar$ plasma was used to produce $\approx 10\,\mu$m wide, $\approx$ 1 cm long, $\approx$ 30 nm deep channels on the Si_3N_4 wafer surface, prior to bonding. This was done to prevent the formation of trapped H_2 bubbles at the bonding interface during the annealing process [12]. The Si_3N_4 wafer was then cleaved into small ($\approx$ 2 cm^2) pieces.

For wafer bonding, the surfaces of the GaAs and Si_3N_4 wafer pieces were cleaned in acetone, then activated in an O_2 plasma for 1 minute, at a pressure of 26.7 Pa (200 mTorr), flow of 1.5×10^{-5} O_2 mol/s (20 sccm) and 200 W radio-frequency power. Pairs of wafers were then placed in contact and pre-bonded under light manual contact. The pre-bonded samples were next annealed at 300 °C for 1 hour in a nitrogen-purged environment to produce a permanent bond. The warm-up and cool-down rates were set to 5 °C/min. At this point, samples consisted of small, rectangular-shaped GaAs wafer pieces permanently bonded onto the surface of larger Si_3N_4 wafers.

We next carefully covered the exposed Si_3N_4 areas on the wafers with Apiezon W wax [23] that had been previously dissolved in trichloethylene (TCE). The dissolved wax wetted the Si_3N_4 surfaces and the sidewalls of the bonded GaAs pieces, however not the exposed back surfaces of the GaAs wafer. We placed the samples on a hotplate at 80 °C for 30 minutes to evaporate the TCE, solidifying the wax. The samples were next immersed in a 3:7 H_3PO_4:H_2O_2 solution for approximately 5 hours, to remove most of the GaAs substrate. They were then transferred to a 4:1 citric acid (50 % mass fraction):H_2O_2 solution, which etched GaAs with a very high selectivity with respect to the AlGaAs sacrificial layers. The samples were left in for approximately 5 hours, until the exposed GaAs wafer surface looked uniform and unchanged. At this point, the GaAs substrate had been completely removed. Next, the samples were dipped in 49 % HF for 30 seconds to remove the AlGaAs sacrifial layers. Finally, the wax was removed with TCE.

Following the wafer bonding step, fabrication proceeded as described in the main text. Further details are provided here. An array of Au alignment marks was first produced on top of the GaAs layer via electron-beam lithography followed by metal-lift-off. A bilayer polymethylmethacrylate/copolymer resist process was used. An electron-beam evaporator was used to deposit a 10 nm Cr adhesion layer, and a 50 nm Au layer. Lift-off was carried out in an acetone bath. Electron-beam lithography with ZEP 520A [23] resist followed by inductively-coupled plasma etching using a Cl_2:Ar chemistry were next used to define GaAs devices aligned to the Au mark array. Because ZEP520A is a positive-tone resist, devices were defined by etching the GaAs only in micron-size areas that surrounded the devices. To remove the remaining GaAs from the rest of the wafer surface, we used a wet-etch approach. First,

e-beam lithography with ma-N 2045negative tone resist [23] was performed to define protection patterns that covered only the device areas and a selected number of Au alignment marks (a protection patch is highlighted in the optical micrograph in Fig. 3b of the main text, covering the GaAs microring resonator and bus waveguide). The samples were then immersed in TFA gold etchant for ≈ 1 min, then in 1020 Cr etch solution for ≈ 1 min. This removed exposed Cr/Au alignment marks, as well as the exposed GaAs layer. The wet etch procedure could be repeated several times without affecting the resist protection layer. Acetone was afterwards used to remove the ma-N resist.

After cleanup of the etched sample surface, a second electron-beam lithography exposure was performed, referenced to the original Au mark array, to define Si_3N_4 waveguide patterns aligned to the previously etched GaAs devices. We emphasize that the alignment marks used were from the original mark array, and were protected during the GaAs wet etch step. Reactive ion etching (RIE) in a CHF_3/CF_4 plasma was used to produce the Si_3N_4 waveguides. The chip was finally cleaved perpendicular to the Si_3N_4 waveguides > 1 mm away from the GaAs devices, to allow access with optical fibers in the endfire configuration.

Supplementary Note 3 - Optimized dipole coupling into the hybrid waveguide

Here we present simulation results for the fundamental TE GaAs mode β-factor of two optimized emission capture structures. In both cases, the active waveguide section is a as shown in Supplementary Figure 2a. The GaAs waveguide has a thickness of 190 nm, and the Si_3N_4 waveguide width is 600 nm. A 100 nm layer of SiO_2 separates the two waveguides. Such a layer can be produced with PECVD, same as the nitride layer grown on our GaAs wafer prior to bonding, without adversely affecting the bond quality. For the first optimized geometry, see Supplementary Figure 2b, the Si_3N_4 thickness is 550 nm, similar to the Si_3N_4 waveguides in our sample. In Supplementary Figure 2b, the Si_3N_4 thickness is 250 nm. In both cases, $\beta > 0.46$ for modes propagating in either $+z$ or $-z$ directions ($2\beta > 0.92$ altogether), for a wavelength range of tens of nanometers around 1100 nm, for GaAs waveguide widths close to 300 nm.

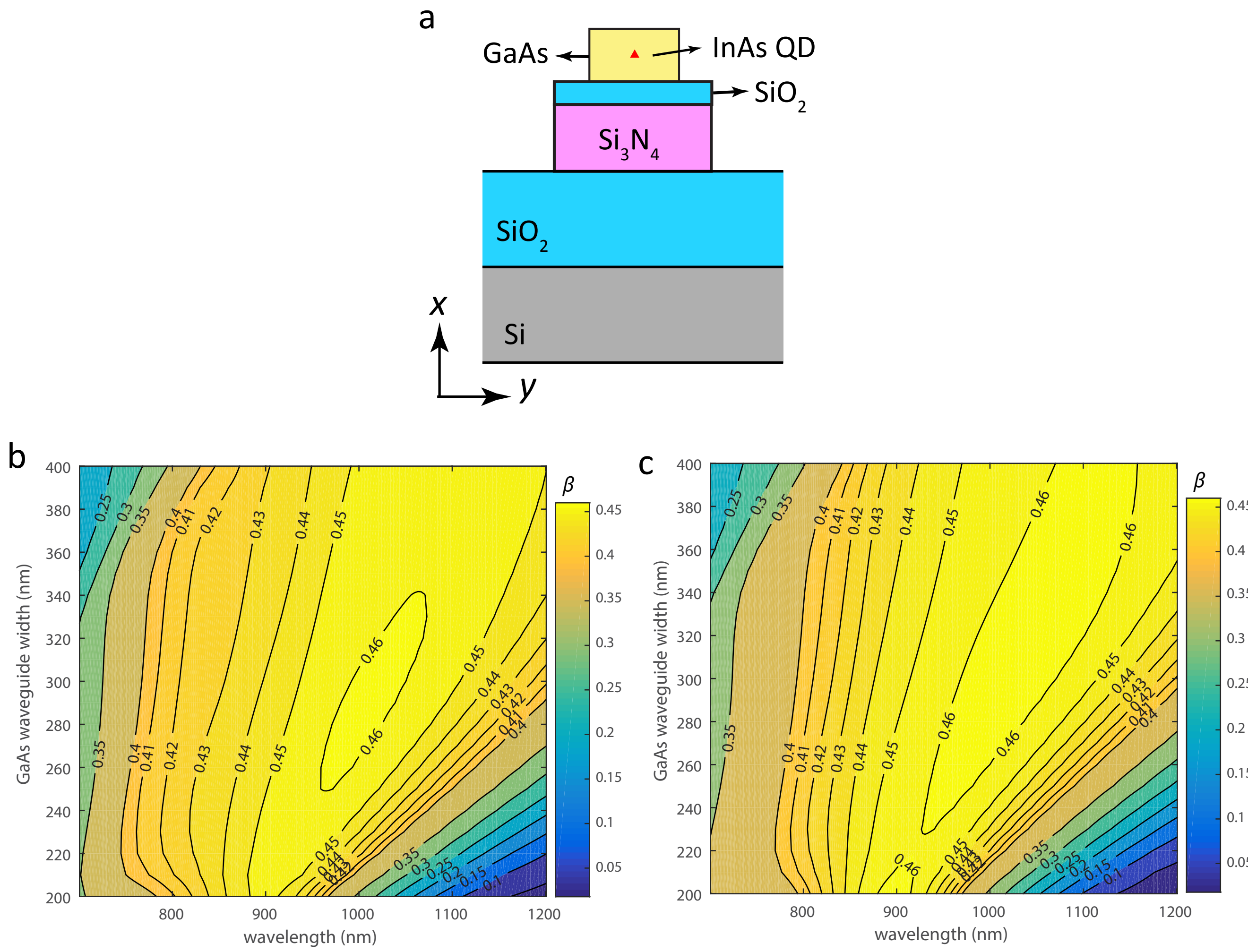

Supplementary Figure 2: **β-factors in optimized structures**. **a** Emission capture structure cross-section. **b**, β factors as a function of wavelength and GaAs waveguide width for the fundamental TE GaAs mode, for a GaAs waveguide with thickness of 190 nm, Si_3N_4 waveguide has a width of 600 nm and thickness of 550 nm. A 100 nm thick layer of SiO_2 separates the Si_3N_4 and GaAs waveguides. The Si_3N_4 dimensions here are relevant for optical nonlinearities, as discussed in the main text. **c**, same as **a** for a 250 nm Si_3N_4 thickness, relevant for linear optics applications.

Supplementary Note 4 - Small mode-volume resonances for strong-coupling regime cavity QED

Here we present simulation results for waveguide-coupled microdisks within our platform, with which the strong-coupling cavity QED regime might be achieved. We first point out that in ref. 24, strong-coupling was achieved in a 2.5 um diameter air-clad microdisk resonator that supported a whispering-gallery mode at $\lambda \approx 1300$ nm, with quality factor $Q \approx 1 \times 10^5$, and standing-wave mode volume $V_{\text{eff}} \approx 3 \times (\lambda/n)^3$. Within our platform, a similar quantum dot-containing GaAs microdisk resonator geometry can be implemented as shown in the schematic of Supplementary Figure 3a. The GaAs microdisk can can be accessed through a GaAs bus waveguide, which is in turn efficiently coupled to the Si_3N_4 waveguide via the mode transformer geometries. The GaAs microdisk contains a single InAs quantum dot, which we next theoretically show can be strongly coupled to a whispering gallery mode of the GaAs/Si_3N_4 microdisk resonator, shown in Supplementary Figure 3b. As in ref. 24, we estimate the coherent coupling rate g_0 between the quantum dot and a standing-wave type whispering-gallery mode in the strong-coupling regime to be

$$g_0 = \frac{1}{2\tau_{\text{sp}}}\sqrt{\frac{3c\tau_{\text{sp}}}{2\pi\lambda V_{\text{eff}}/(\lambda/n)^3}}, \tag{1}$$

where τ_{sp} is the natural quantum dot spontaneous emission lifetime, n is the GaAs refractive index, λ is the wavelength of the whispering-gallery mode. Supplementary Figure 3b shows a cross-sectional field amplitude map of the azimuthal $m = 18$ whispering-gallery mode for a hybrid GaAs microdisk of height 200 nm and diameter 2 μm within our platform, calculated with the finite-element method. The hybrid resonator consists of a GaAs microdisk that sits on top of a 250 nm thick Si_3N_4 cylinder of radius 1.2 μm, over a SiO_2 substrate. The calculated radiation-limited quality factor here is $Q_{\text{rad}} \approx 10^9$, and the standing-wave mode volume is $V_{\text{eff}} \approx 6(\lambda/n)^3$. Using this value in Eq. (1), together with the typical parameters $\tau_{\text{sp}} = 1$ ns, $n = 3.4$ and $\lambda = 937$ nm for InAs quantum dots, we obtain $g_0/2\pi \approx 13$ GHz. With this, the strong coupling regime can be within reach for realistic quantum dot linewidths (< 1 GHz) and coupled-cavity quality factors, $Q \approx 1 \times 10^4$ (corresponding to a cavity decay rate $\kappa \approx 6$ GHz).

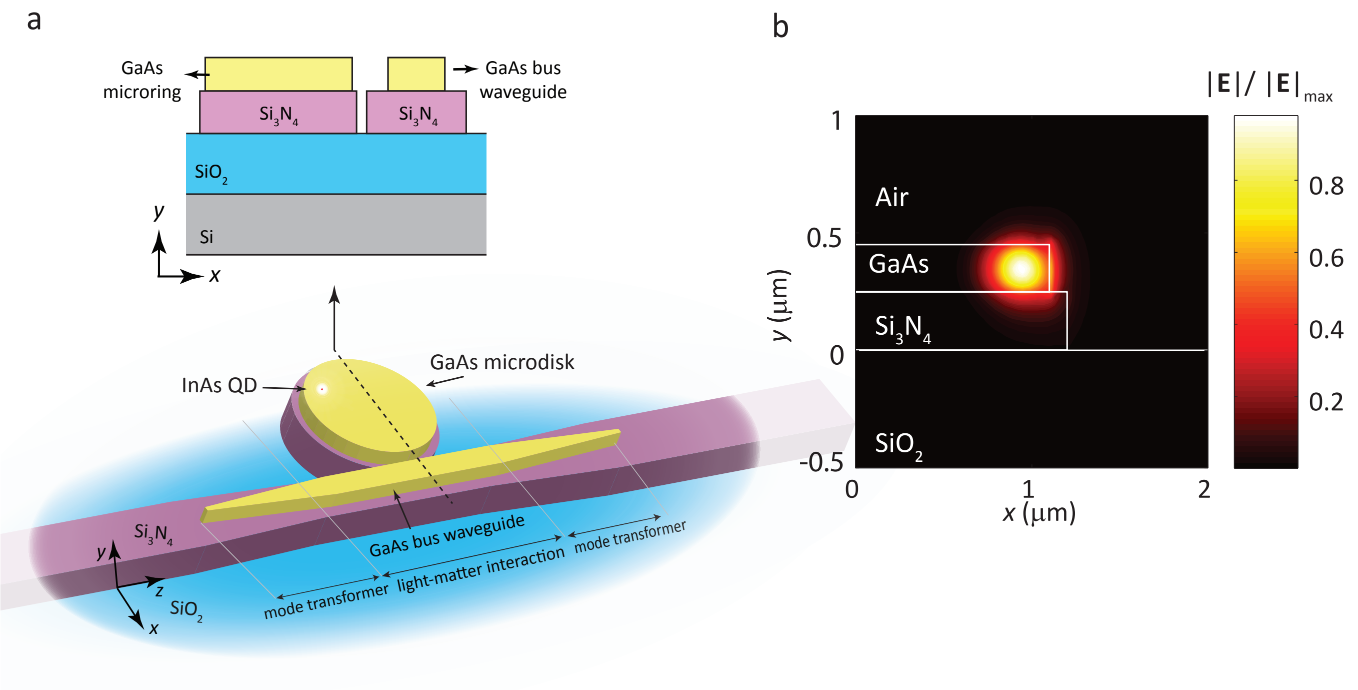

Supplementary Figure 3: **Hybrid microdisk whispering-gallery modes for strong coupling**. **a**, Schematic of waveguide-coupled hybrid microdisk resonator within out platform. The top image shows a cross-section of the geometry, corresponding to the dashed line in the 3D schematic. **b**, Cross-sectional electric field amplitude profile for the $m = 18$ azimuthal whispering-gallery hybrid microdisk mode, which has a standing wave mode volume of $6(\lambda/n)^3$ and a predicted vacuum coupling strength of 13 GHz to a single, optimally aligned quantum dot.

We note that although a microdisk geometry was chosen in this example, we expect very similar results for similar-sized microrings, which support nearly identical modes (though they interact with two sidewalls and are laterally

offset with respect to the microdisk modes). Moreover, our experimentally-demonstrated microrings already exhibit $Q \approx 2 \times 10^4$ (Fig. 6a). Strong coupling in these devices should then be achievable if this Q can be maintained when the microring diameter is shrunk to ≈ 2 μm, and if the quantum dot location with respect to the field maximum can be precisely set. Finally, we note that the refractive index contrast between GaAs and Si_3N_4 is large enough that we expect that other microcavity geometries, such as photonic crystals, may also be able to reach the strong coupling regime in our platform.

Supplementary Note 5 - Waveguide propagation loss estimate

To estimate propagation losses in our Si_3N_4 waveguides, we measure the transmission spectrum of a microring resonator fabricated on the same material using the same lithography and etching processes. The microring is formed by a waveguide with a $\approx$ 800 nm width and has a radius $R = 50$ μm. It is coupled evanescently to an 800 nm wide Si_3N_4 bus waveguide, which is in turn coupled-to with lensed optical fibers in the endfire configuration. The transmission spectrum shown in Supplementary Figure 4 is obtained by scanning the wavelength of a probing external-cavity tunable laser within the 940 nm band. The experimental data is fitted with the function

$$T(\omega) = \left| 1 - \frac{\kappa_{\rm e}/2}{\kappa/2 + i\,(\omega - \omega_0)} \right|^2, \tag{2}$$

where κ is the total cavity loss rate, $\kappa_{\rm e}$ is the external coupling rate between the cavity and the bus waveguide, ω_0 is the cavity angular frequency. Defining the total and coupling quality factors $Q = \omega/\kappa$ and $Q_{\rm e} = \omega/\kappa_{\rm e}$, the intrinsic quality factor $Q_{\rm i}$, associated with the intrinsic loss rate $\kappa_{\rm i}$ of the microring, is $Q_{\rm i} = \omega/\kappa_{\rm i} = \left(Q^{-1} - Q_{\rm e}^{-1}\right)^{-1}$. Fitting the spectrum in Supplementary Figure 4 gives $Q \approx 5 \times 10^5$, $Q_{\rm e} \approx 3 \times 10^6$, $Q_{\rm i} \approx 6 \times 10^5$. Such high quality factors are already comparable to those of similar Si_3N_4 microring devices recently used for demonstrating efficient on-chip wavelength conversion of single-photon level systems using the four-wave-mixing Bragg-scattering (FWM-BS) $\chi^{(3)}$ nonlinear optical process [7], and is sufficient for the realization of narrow-band on-chip filters for quantum dot emission, as demonstrated in [25]. For $\exp(-\alpha \cdot 2\pi R) \approx 1$, where α are the waveguide propagation losses, we may write [26] $\alpha \approx \lambda/(Q \cdot R \cdot \mathrm{FSR})$, where FSR=1.3 nm is the free-spectral range of our microring. With this, we estimate $\alpha \approx 1.1$ dB/cm. Such a loss figure is considerably lower then the lowest reported to date for GaAs waveguides used in single quantum dot investigations ($\approx$ 15 dB/cm in the 900 nm band in ref. 2, $\approx$ 17 dB/cm in the 1300 nm band [5]), and is lower than the $\approx$ 2.5 dB/cm-4 dB/cm observed in the 900 nm band in the SiN waveguides of ref. 19. It is worth mentioning that losses of the order of 1 dB/cm have only been reported for GaAs waveguides at 1330 nm [27] and 1550 nm wavelengths [28] (far away from the GaAs absoption edge at $\approx$ 800 nm), in purely passive ridge geometries supporting modes with weak lateral confinement. Weak confinement translates into large effective modal areas, which in turn lead to lesser light-matter interaction strengths - essentially geometries that are not ideal for coupling to single quantum dots.

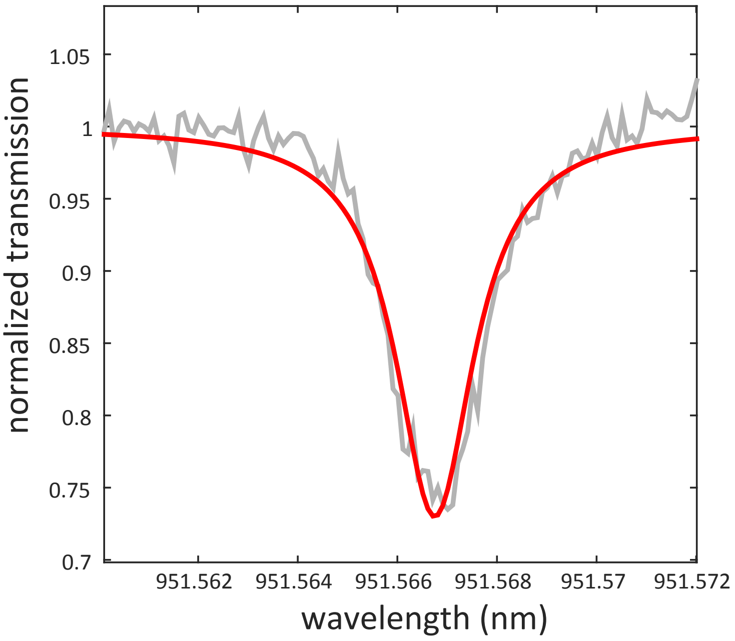

Supplementary Figure 4: **Si_3N_4 waveguide loss estimate**. Transmission spectrum for a waveguide-coupled Si_3N_4 microring resonator of 800 nm width and radius $R = 50$ μm. Gray curve: experimental data; red curve: fit. The fit gives a loaded $Q \approx 5 \times 10^5$, coupling $Q_{\rm e} \approx 3 \times 10^6$, and intrinsic $Q_{\rm i} \approx 6 \times 10^5$.

Supplementary Note 6 - Cryogenic measurement experimental setup

The setup used for low temperature cryogenic measurements is shown in Supplementary Figure 5. Samples were placed on a fixed mount inside a liquid Helium flow cryostat and cooled down ≈ 7 K. A microscope consisting of a long-working distance objective (20×, NA=0.4), beamsplitter (BS) and combination zoom lens / illuminator system mounted at the top cryostat window allowed devices on the sample surface to be imaged. An example image of a GaAs microring resonator device can be seen in the "sample imaging" box in the schematic of Supplementary Figure 5. Light from an external cavity tunable laser (ECDL) with center wavelength around 1060 nm was introduced into the objective via the beamsplitter, to produce a small (≈ 5 μm) spot that pumped QDs at select locations on the device under test. As discussed in the main text, photoluminescence from the QDs was coupled to Si_3N_4 waveguides, and collected at the cleaved edge of the chip with a lensed fiber. A nanopositiong stage stack placed inside the cryostat allowed the lensed fibers to be aligned to waveguide facets, as illustrated in Supplementary Figure 5.

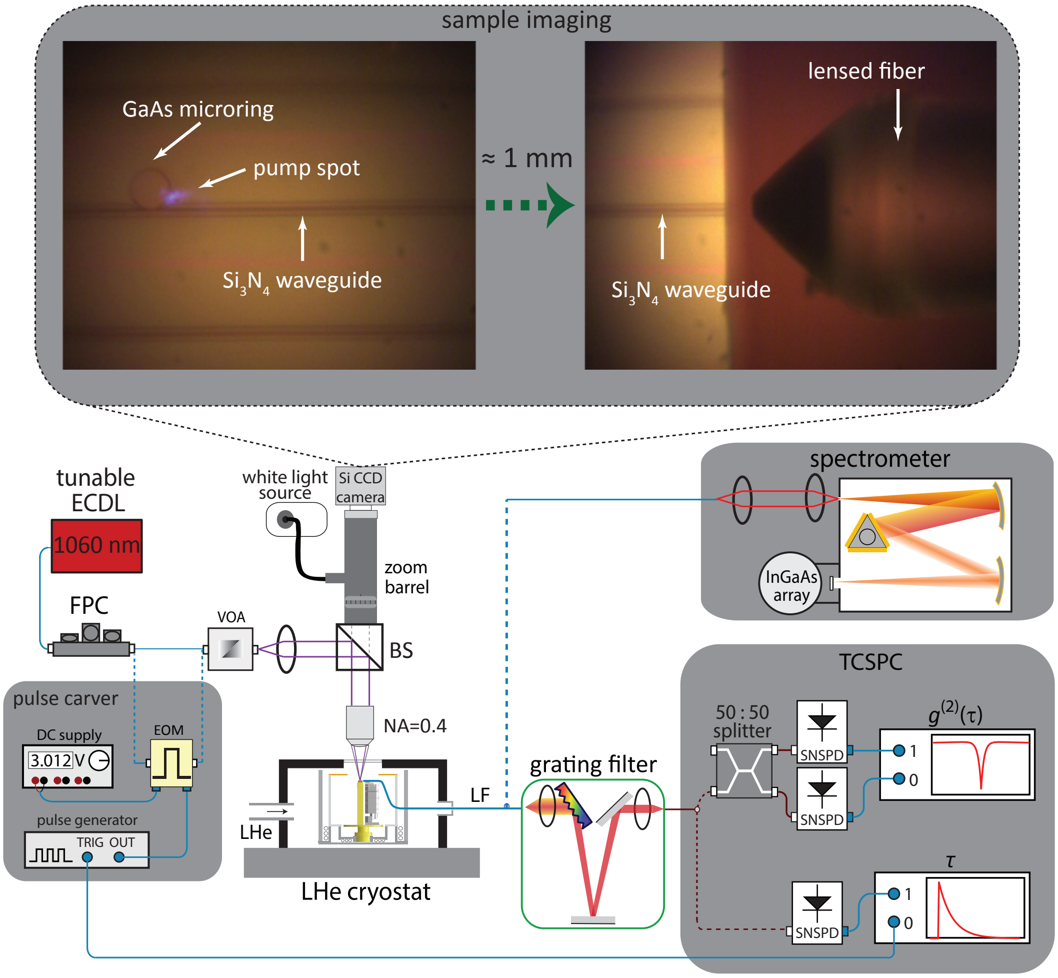

Supplementary Figure 5: **Cryogenic measurement setup**. Devices are tested inside a liquid Helium flow cryostat. A window at the top allows imaging of the sample, as shown in the "sample imaging" box. Devices are also pumped from the top with laser light (purple spot over portion of the GaAs microring resonator), and quantum dot photoluminescence is coupled into the Si_3N_4 waveguide. The emitted light travels ≈ 1 mm down the Si_3N_4 waveguide until the cleaved edge of the chip, where it is collected with a lensed fiber mounted to a nanopositioning stage inside the cryostat. The collected light can then be routed to a grating spectrometer, or filtered in a grating filter and then routed towards time-correlated single-photon counting measurements. Pulsed pumping can be achieved by modulating continuous-wave laser light with an electro-optic modulator. ECDL: external-cavity diode laser; FPC: fiber polarization controller; EOM: electro-optic modulator; BS: beamsplitter; VOA: variable optical attenuator. LF: lensed fiber. SNSPD: superconducting nanowire single-photon detector; TCSPC: time-correlated single-photon counting; CCD: charge-coupled device; LHe: liquid Helium

The collected photoluminescence was either be routed to a grating spectrometer equipped with a liquid-nitrogen cooled InGaAs photodiode array, or filtered through a $\approx$ 700 pm bandpass tunable grating filter and then routed towards our single-photon detection system for time-correlated single-photon counting (TCSPC) measurements of excited state lifetime and second-order correlations. For correlation measurements, we used a Hanbury-Brown and Twiss setup consisting of a fiber-based 50:50 beamsplitter connected to two amorphous WSi superconducting nanowire single-photon detectors (SNSPDs) [29]. Detector counts were correlated in a TCSPC unit.

For lifetime measurements, a 10 GHz lithium niobate electro-optic modulator (EOM) was used to produce a 80 MHz, $\approx$ 200 ps pulse train from the CW ECDL laser [30]. A fiber-based polarization controller (FPC) was used to control the polarization of the ECDL light going into the EOM, and a DC bias was applied to the EOM to maximize signal extinction. An electrical pulsed source was used to produce an 80 MHz train of $\approx$ 200 ps pulses of $<$ 1 V peak amplitude, which was then amplified and used to drive the EOM modulator via its radio frequency (RF) port. A trigger signal from the pulse generator served as the reference channel in our TCSPC system. Supplementary Figure 6a shows a typical temporal profile for the pulses produced by the EOM, detected with an SNSPD. Pulse FWHM of $\approx$ 200 ps and $>$ 20 dB extinction are observed. The pulsed electrical signal produced small satellite peaks that were imprinted in the optical signal, as indicated in Supplementary Figure 6a. Impulse response functions (IRFs) such as shown in Supplementary Figure 6a were used in decay lifetime fits as explained below, so that the effect of satellite peaks, though minimal, was accounted for.

To determine the time resolution of our detection system, we launched attenuated, few-picosecond pulses from a Ti:Sapphire mode-locked laser at 975 nm into the SNSPDs, to obtain the temporal trace in Supplementary Figure 6b. The peak can be well fitted with a Gaussian with standard deviation σ = 129 ps $\pm$ 0.04 ps (uncertainty is a 95 % least-squares fit confidence interval, corresponding to two standard deviations).

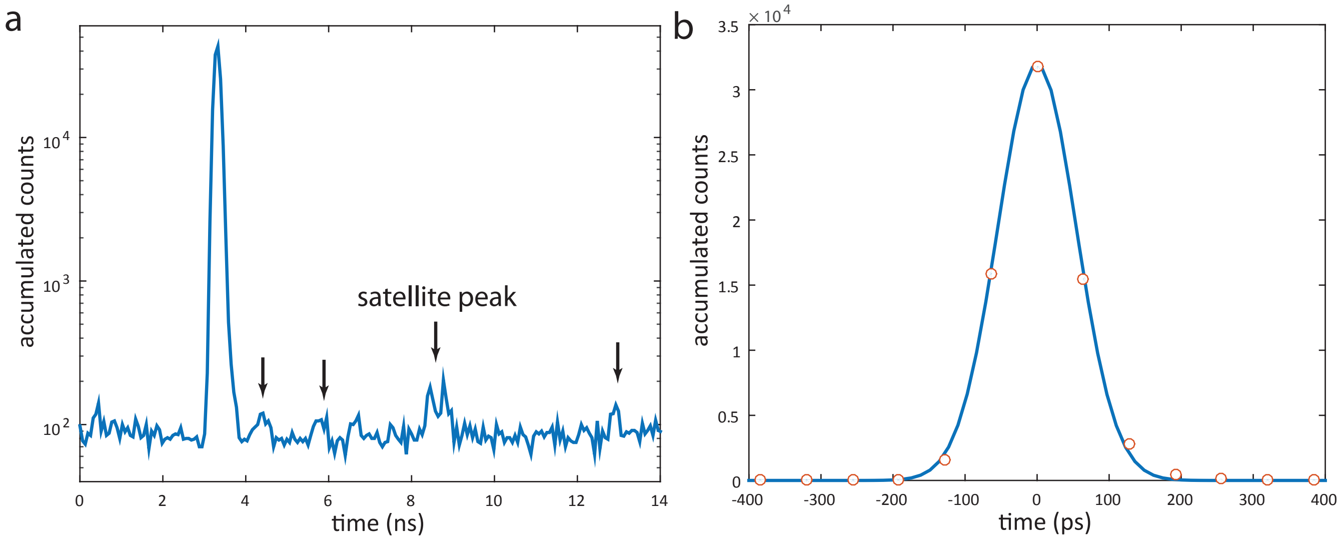

Supplementary Figure 6: **Impulse response functions**. **a**, Temporal profile of pulsed pump achieved with an electro-optic modulator (EOM). Satellite peaks due to an imperfect electrical driving signal are pinpointed with arrows. **b**, Impulse response of the SNSPD-based detection system, obtained with a $\approx$ ps pulsed Ti:Sapphire laser source.

Supplementary Note 7 - Purcell enhancement in the heterogeneous platform

Here we provide further information about the Purcell enhancement data and fits for the quantum dot in a microring resonator presented in Figs. 6d-6g of the main text. Supplementary Figures 7a and 7b respectively show lifetimes and corresponding decay component amplitudes for the fits, as a function of the detuning Δ. It is apparent that the fast lifetimes vary considerably, from $\approx$ 414 ps at $\Delta \approx -0.26$ nm to $\approx$ 263 ps at $\Delta \approx -0.07$ nm, then to $\approx$ 1.1 ns at $\Delta = 0.84$ nm. Slow lifetimes remain consistently above 1 ns. The fast decay contribution remains above 50 % for all detunings except $\Delta \approx 0.36$ nm, where a second exciton (X_2) is seen to couple to the same whispering-gallery mode in Fig. 6f. The contributions of the two excitons and the cavity to the detected signal in the lifetime measurements is estimated through fits to the emission spectra at each detuning, shown in Supplementary Figure 7c. The X_1 contribution is seen to be dominant everywhere (except $\Delta \approx 0.36$ nm). The X_2 contribution is maximized at $\Delta \approx 0.36$ nm, but remains below 0.015 everywhere. These results indicate that the fast lifetimes can be assigned to the X_1 exciton. Further supporting this assignment is the fact that the good quality of the $g^{(2)}(\tau)$ fit in Fig. 6e was achieved by including a Poissonian background equal to the cavity contribution in the PL spectrum fit of Fig. 6d. Uncertainties for $g^{(2)}(0)$, Δ and the X_1, X_2 and cavity PL contributions are 95 % fit confidence intervals (two standard deviations). Uncertainties for τ are single standard deviations from the exponential decay fit procedure.

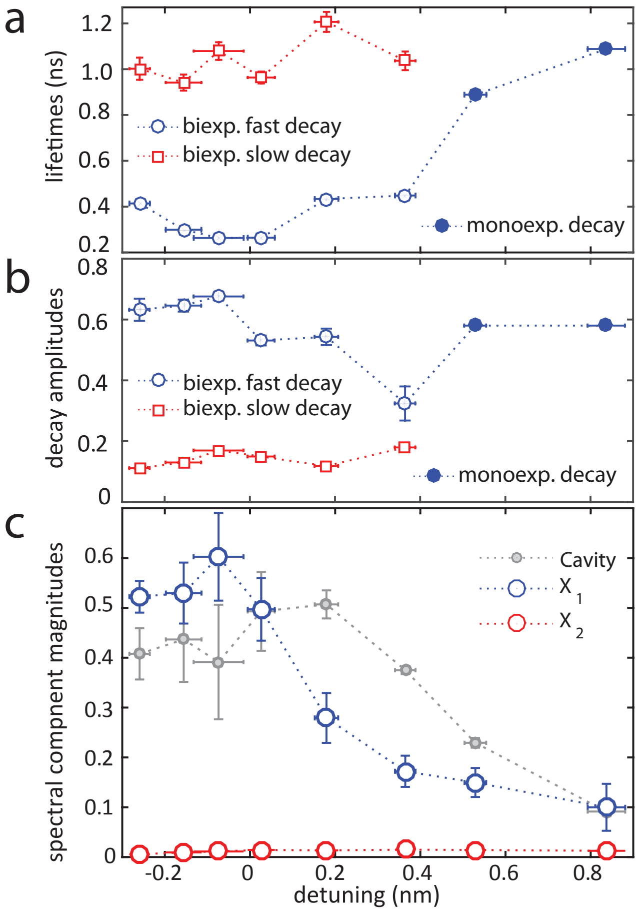

Supplementary Figure 7: **Lifetime and PL intensity fit results**. **a**, Decay lifetimes for the fits in Fig. 6f, as a function of the detuning Δ. Open blue circles and red squares are the fast and slow biexponential decay lifetimes, closed blue circles are the monoexponential decay lifetimes. **b**, Corresponding amplitudes for the different decay components. **c**, magnitudes of the X_1, X_2 and cavity Lorentzians used to fit the PL spectra in **f**, normalized to the sum of the integrated intensities of the three components before filtering. Uncertainties for Δ and the X_1, X_2 and cavity PL contributions are 95 % fit confidence intervals (two standard deviations). Uncertainties for τ are single standard deviations from the exponential decay fit procedure.

Supplementary Note 8 - Fisher information matrix

Variances for the estimated decay lifetime parameters in $\boldsymbol{\theta}$ can be obtained from the diagonal elements of the inverse of the Fisher Information Matrix $M(\theta)$ with

$$[M(\boldsymbol{\theta})]_{mn} = \mathbb{E}_{\boldsymbol{\theta}}\left[\frac{\partial \ln f_{\boldsymbol{\theta}}^{(k)}(Y^k)}{\partial \theta_m}\frac{\partial \ln f_{\boldsymbol{\theta}}^{(k)}(Y^k)}{\partial \theta_n}\right]. \tag{3}$$

Here, $\mathbb{E}_{\boldsymbol{\theta}}$ stands for expectation value, and $f_{\boldsymbol{\theta}}^{(k)}(y^k)$ is the probability mass function for the counts in each bin of the liftetime trace, which constitute a sequence of random variables with multinomial distibution [31]:

$$f_{\boldsymbol{\theta}}^{(k)}(y^k) = \frac{N!}{\prod_{i=1}^{k} y_i!}\prod_{i=1}^{k} p_i^{y_i}(\boldsymbol{\theta}),\ \sum_{i=1}^{k} y_i = N,\ \sum_{i=1}^{k} p_i(\boldsymbol{\theta}) = 1 \tag{4}$$

Noting that

$$\frac{\partial \ln f_{\boldsymbol{\theta}}^{(k)}(y^k)}{\partial \theta_m} = \sum_{i=1}^{k}\frac{y_i}{p_i(\boldsymbol{\theta})}\frac{\partial p_i(\boldsymbol{\theta})}{\partial \theta_m}, \tag{5}$$

we can write

$$\sum_{i=1}^{k}\sum_{j=1}^{k}\frac{\mathbb{E}_{\boldsymbol{\theta}}\left[Y_iY_j\right]}{p_i(\boldsymbol{\theta})p_j(\boldsymbol{\theta})}\frac{\partial p_i(\boldsymbol{\theta})}{\partial \theta_m}\frac{\partial p_j(\boldsymbol{\theta})}{\partial \theta_n} \tag{6}$$

Now

$$\mathbb{E}_{\boldsymbol{\theta}}\left[Y_iY_j\right] = \begin{cases} \mathrm{var}_{\boldsymbol{\theta}}\left[Y_i\right] + \left(\mathbb{E}_{\boldsymbol{\theta}}\left[Y_i\right]\right)^2 = (N^2-N)p_i^2(\boldsymbol{\theta}) + Np_i(\boldsymbol{\theta}) & \text{if } i=j, \\ \mathrm{COV}_{\boldsymbol{\theta}}\left[Y_i,Y_j\right] + \mathbb{E}_{\boldsymbol{\theta}}\left[Y_i\right]\mathbb{E}_{\boldsymbol{\theta}}\left[Y_j\right] = (N^2-N)p_i(\boldsymbol{\theta})p_j(\boldsymbol{\theta}) & \text{if } i\neq j, \end{cases} \tag{7}$$

where $\mathrm{var}_{\boldsymbol{\theta}}$ and $\mathrm{COV}_{\boldsymbol{\theta}}$ are the variance and the covariance operators with respect to $\boldsymbol{\theta}$, respectively. Substituting (7) into (6), it follows that

$$\begin{aligned}\mathbb{E}_{\boldsymbol{\theta}}\left[\frac{\partial \ln f_{\boldsymbol{\theta}}^{(k)}(Y^k)}{\partial \theta_m}\frac{\partial \ln f_{\boldsymbol{\theta}}^{(k)}(Y^k)}{\partial \theta_n}\right] =& \sum_{i=1}^{k}\frac{(N^2-N)p_i^2(\boldsymbol{\theta})y + Np_i(\boldsymbol{\theta})}{p_i^2(\boldsymbol{\theta})}\frac{\partial p_i(\boldsymbol{\theta})}{\partial \theta_m}\frac{\partial p_i(\boldsymbol{\theta})}{\partial \theta_n} \\ &+ \underbrace{\sum_{i=1}^{k}\sum_{j=1}^{k}}_{i\neq j}\frac{(N^2-N)p_i(\boldsymbol{\theta})p_j(\boldsymbol{\theta})y}{p_i(\boldsymbol{\theta})p_j(\boldsymbol{\theta})}\frac{\partial p_i(\boldsymbol{\theta})y}{\partial \theta_m}\frac{\partial p_j(\boldsymbol{\theta})}{\partial \theta_n}\end{aligned} \tag{8}$$

The first summation can be written as

$$\sum_{i=1}^{k}\frac{(N^2-N)p_i^2(\boldsymbol{\theta}) + Np_i(\boldsymbol{\theta})}{p_i^2(\boldsymbol{\theta})}\frac{\partial p_i(\boldsymbol{\theta})}{\partial \theta_m}\frac{\partial p_i(\boldsymbol{\theta})}{\partial \theta_n} = (N^2-N)\sum_{i=1}^{k}\frac{\partial p_i(\boldsymbol{\theta})y}{\partial \theta_m}\frac{\partial p_i(\boldsymbol{\theta})}{\partial \theta_n} + N\sum_{i=1}^{k}\frac{1}{p_i(\boldsymbol{\theta})}\frac{\partial p_i(\boldsymbol{\theta})y}{\partial \theta_m}\frac{\partial p_i(\boldsymbol{\theta})}{\partial \theta_n}. \tag{9}$$

The second summation can be written as

$$\underbrace{\sum_{i=1}^{k}\sum_{j=1}^{k}}_{i\neq j}\frac{(N^2-N)p_i(\boldsymbol{\theta})p_j(\boldsymbol{\theta})}{p_i(\boldsymbol{\theta})p_j(\boldsymbol{\theta})}\frac{\partial p_i(\boldsymbol{\theta})}{\partial \theta_m}\frac{\partial p_j(\boldsymbol{\theta})}{\partial \theta_n} = (N^2-N)\underbrace{\sum_{i=1}^{k}\sum_{j=1}^{k}}_{i\neq j}\frac{\partial p_i(\boldsymbol{\theta})}{\partial \theta_m}\frac{\partial p_j(\boldsymbol{\theta})y}{\partial \theta_n} \tag{10}$$

Substituting (9) and (10) into (8), it follows that

$$\mathbb{E}_{\boldsymbol{\theta}}\left[\frac{\partial \ln f_{\boldsymbol{\theta}}^{(k)}(Y^k)}{\partial \theta_m}\frac{\partial \ln f_{\boldsymbol{\theta}}^{(k)}(Y^k)}{\partial \theta_n}\right] = (N^2-N)\sum_{i=1}^{k}\sum_{j=1}^{k}\frac{\partial p_i(\boldsymbol{\theta})}{\partial \theta_m}\frac{\partial p_j(\boldsymbol{\theta})}{\partial \theta_n} + N\sum_{i=1}^{k}\frac{1}{p_i(\boldsymbol{\theta})}\frac{\partial p_i(\boldsymbol{\theta})y}{\partial \theta_m}\frac{\partial p_i(\boldsymbol{\theta})}{\partial \theta_n}. \tag{11}$$

Noting that

$$\sum_{i=1}^{k}\sum_{j=1}^{k}\frac{\partial p_i(\boldsymbol{\theta})}{\partial\theta_m}\frac{\partial p_j(\boldsymbol{\theta})}{\partial\theta_n}=\sum_{i=1}^{k}\frac{\partial p_i(\boldsymbol{\theta})y}{\partial\theta_m}\sum_{j=1}^{k}\frac{\partial p_j(\boldsymbol{\theta})}{\partial\theta_n}=\frac{\partial\left(\sum_{i=1}^{k}p_i(\boldsymbol{\theta})\right)}{\partial\theta_m}\frac{\partial\left(\sum_{j=1}^{k}p_j(\boldsymbol{\theta})\right)}{\partial\theta_n}=\frac{\partial(1)}{\partial\theta_m}\frac{\partial(1)}{\partial\theta_n}=0, \tag{12}$$

it follows that

$$\mathbb{E}_{\boldsymbol{\theta}}\left[\frac{\partial\ln f_{\boldsymbol{\theta}}^{(k)}(Y^k)}{\partial\theta_m}\frac{\partial\ln f_{\boldsymbol{\theta}}^{(k)}(Y^k)}{\partial\theta_n}\right]=N\sum_{i=1}^{k}\frac{1}{p_i(\boldsymbol{\theta})y}\frac{\partial p_i(\boldsymbol{\theta})}{\partial\theta_m}\frac{\partial p_i(\boldsymbol{\theta})}{\partial\theta_n}. \tag{13}$$

We next define

$$p_i(\tau)=e^{-ir/k}\frac{e^{\frac{r}{k}}-1}{1-e^{-r}},\ r\triangleq\frac{i\cdot\Delta t}{\tau} \tag{14}$$

$$\frac{\partial p_i(\tau)}{\partial\tau}=\frac{-r}{\tau}p_i(\tau)\left(-\frac{i}{k}+\frac{e^{r/k}}{(e^{r/k}-1)k}-\frac{e^{-r}}{1-e^{-r}}\right). \tag{15}$$

For a monoexponential decay when a portion b of the signal is due to background emission,

$$p_i(\tau,b)=\frac{b}{k}+(1-b)p_i(\tau) \tag{16}$$

The Fisher matrix in this case can be computed with eq.(13) and

$$\frac{\partial p_i(\tau,b)}{\partial b}=\frac{1}{k}-p_i(\tau) \tag{17}$$

$$\frac{\partial p_i(\tau,b)}{\partial\tau}=(1-b)\frac{-r}{\tau}p_i(\tau)\left(-\frac{i}{k}+\frac{e^{r/k}}{(e^{r/k}-1)k}-\frac{e^{-r}}{1-e^{-r}}\right) \tag{18}$$

For biexponential decay with a background, let $\tau\triangleq(\tau_1,\tau_2)^T$. Then $p_i(\tau,a,b)$ (where a is the contribution of the first exponential decay) may be expressed as

$$p_i(\tau,b,a)=\frac{b}{k}+(1-b)\left[ap_i(\tau_1)+(1-a)p_i(\tau_2)\right] \tag{19}$$

The Fisher matrix in this case can be computed with eq.(13) and

$$\frac{\partial p_i(\tau,b,a)}{\partial b}=\frac{1}{k}-\left[ap_i(\tau_1)+(1-a)p_i(\tau_2)\right] \tag{20}$$

$$\frac{\partial p_i(\tau,b,a)}{\partial\tau_1}=a(1-b)\frac{\partial p_i(\tau_1)}{\partial\tau_1} \tag{21}$$

$$\frac{\partial p_i(\tau,b,a)}{\partial\tau_2}=(1-a)(1-b)\frac{\partial p_i(\tau_2)}{\partial\tau_2} \tag{22}$$

$$\frac{\partial p_i(\tau,b,a)}{\partial a}=(1-b)\left(p_i(\tau_1)-p_i(\tau_2)\right). \tag{23}$$

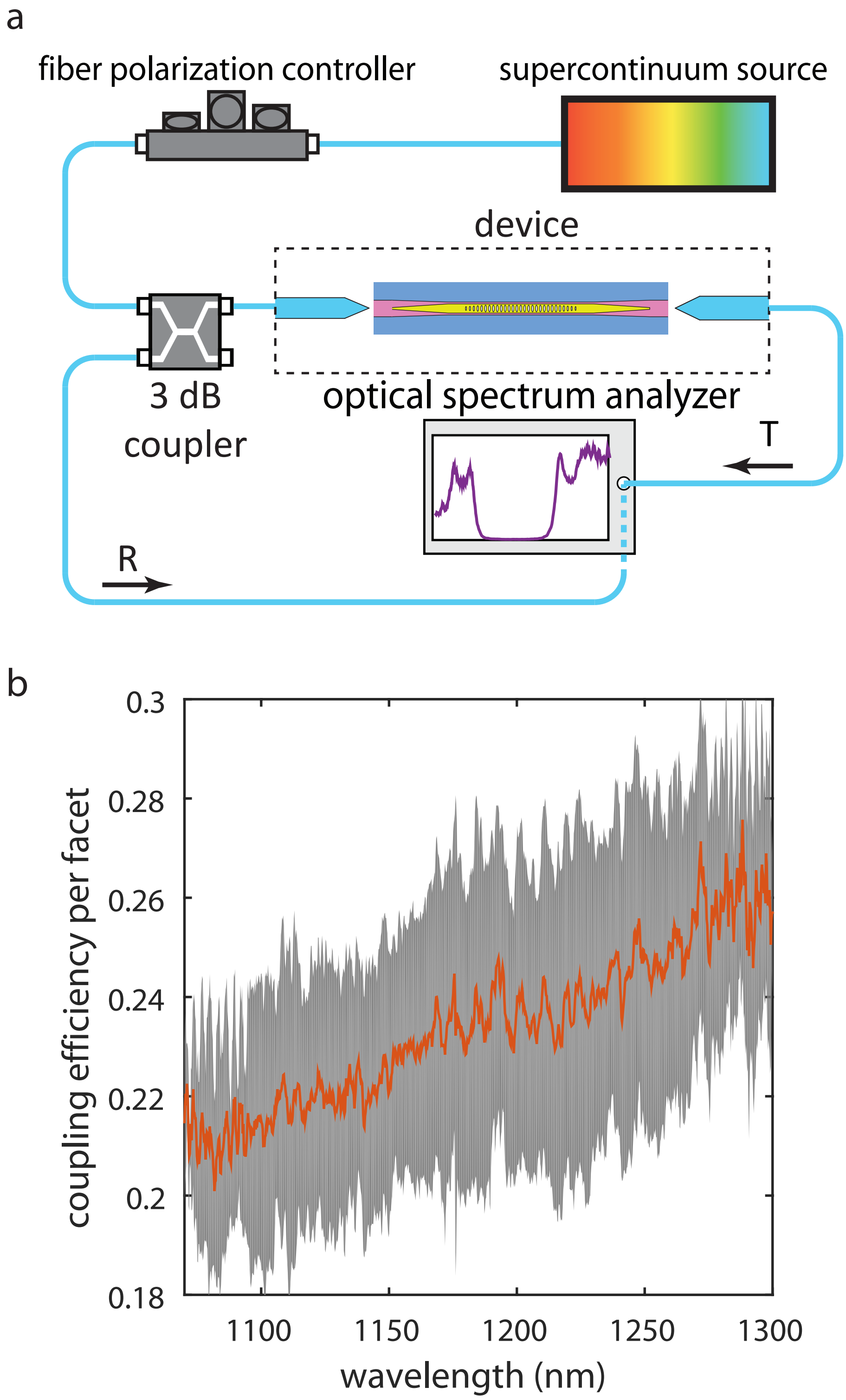

Supplementary Figure 8: **Photonic characterization setup**. **a**, Experimental setup for passive, endfire coupling measurement of transmitted (T) and reflected (R) power. **b**, Measured coupling efficiency per facet (including fiber-to-waveguide coupling and propagation losses in the waveguide between the facet and a GaAs device) as a function of wavelength. The red curve and grey area are the mean value and standard deviation over three measured waveguides.

Supplementary References

[1] C. P. Dietrich, A. Fiore, M. G. Thompson, M. Kamp, and S. Höfling, "GaAs integrated quantum photonics: Towards compact and multi-functional quantum photonic integrated circuits," Laser & Photonics Reviews **10**, 870-894 (2016).
[2] K. D. Jöns, U. Rengstl, M. Oster, F. Hargart, M. Heldmaier, S. Bounouar, S. M. Ulrich, M. Jetter, and P. Michler, "Monolithic on-chip integration of semiconductor waveguides, beamsplitters and single-photon sources," Journal of Physics D: Applied Physics **48**, 085 101 (2015).
[3] G. Reithmaier, M. Kaniber, F. Flassig, S. Lichtmannecker, K. Müller, A. Andrejew, J. Vučković, R. Gross, and J. J. Finley, "On-Chip Generation, Routing, and Detection of Resonance Fluorescence," Nano Lett. **15**, 5208–5213 (2015).
[4] N. Prtljaga, R. J. Coles, J. O'Hara, B. Royall, E. Clarke, A. M. Fox, and M. S. Skolnick, "Monolithic integration of a quantum emitter with a compact on-chip beam-splitter," Applied Physics Letters **104**, 231 107 (2014).
[5] S. F. poor, T. B. Hoang, L. Midolo, C. P. Dietrich, L. H. Li, E. H. Linfield, J. F. P. Schouwenberg, T. Xia, F. M. Pagliano, F. W. M. v. Otten, and A. Fiore, "Efficient coupling of single photons to ridge-waveguide photonic integrated circuits," Applied Physics Letters **102**, 131 105 (2013).
[6] M. Davanço and K. Srinivasan, "Fiber-coupled semiconductor waveguides as an efficient optical interface to a single quantum dipole," Opt. Lett. **34**, 2542–2544 (2009).
[7] Q. Li, M. Davanço, and K. Srinivasan, "Efficient and low-noise single-photon-level frequency conversion interfaces using silicon nanophotonics," Nature Photonics **10**, 406–414 (2016).
[8] T. Shoji, T. Tsuchizawa, T. Watanabe, K. Yamada, and H. Morita, "Low loss mode size converter from 0.3 μm square Si wire waveguides to singlemode fibres," Electronics Letters **38**, 1669–1670 (2002).
[9] T. Tsuchizawa, K. Yamada, H. Fukuda, T. Watanabe, J.-i. Takahashi, M. Takahashi, T. Shoji, E. Tamechika, S. Itabashi, and H. Morita, "Microphotonics devices based on silicon microfabrication technology," IEEE Journal of Selected Topics in Quantum Electronics **11**, 232–240 (2005).
[10] X. Sun and A. Yariv, "Engineering supermode silicon/III-V hybrid waveguides for laser oscillation," JOSA B **25**, 923–926 (2008).
[11] M. Davanço and K. Srinivasan, "Efficient spectroscopy of single embedded emitters using optical fiber taper waveguides," Optics Express **17**, 10 542–10 563 (2009).
[12] A. W. Fang, H. Park, Y.-h. Kuo, R. Jones, O. Cohen, D. Liang, O. Raday, M. N. Sysak, M. J. Paniccia, and J. E. Bowers, "Hybrid silicon evanescent devices," Materials Today **10**, 28–35 (2007).
[13] J. E. Bowers, T. Komljenovic, M. Davenport, J. Hulme, A. Y. Liu, C. T. Santis, A. Spott, S. Srinivasan, E. J. Stanton, and C. Zhang, "Recent advances in silicon photonic integrated circuits," p. 977402 (2016).
[14] B. Rigal, C. Jarlov, P. Gallo, B. Dwir, A. Rudra, M. Calic, and E. Kapon, "Site-controlled quantum dots coupled to a photonic crystal molecule," Applied Physics Letters **107**, 141 103 (2015).
[15] C. Schneider, A. Huggenberger, M. Gschrey, P. Gold, S. Rodt, A. Forchel, S. Reitzenstein, S. Höfling, and M. Kamp, "In(Ga)As/GaAs site-controlled quantum dots with tailored morphology and high optical quality," physica status solidi (a) **209**, 2379–2386 (2012).
[16] M. Helfrich, P. Schroth, D. Grigoriev, S. Lazarev, R. Felici, T. Slobodskyy, T. Baumbach, and D. M. Schaadt, "Growth and characterization of site-selective quantum dots," physica status solidi (a) **209**, 2387–2401 (2012).
[17] L. Sapienza, M. Davanço, A. Badolato, and K. Srinivasan, "Nanoscale optical positioning of single quantum dots for bright and pure single-photon emission," Nature Communications **6**, 7833 (2015).
[18] J. Liu, M. I. Davanco, L. Sapienza, K. Konthasinghe, J. V. D. M. Cardoso, J. D. Song, A. Badolato, and K. Srinivasan, "Cryogenic photoluminescence imaging system for nanoscale positioning of single quantum emitters," Review of Scientific Instruments **88**, 023 116 (2017).
[19] I. E. Zadeh, A. W. Elshaari, K. D. Jöns, A. Fognini, D. Dalacu, P. J. Poole, M. E. Reimer, and V. Zwiller, "Deterministic Integration of Single Photon Sources in Silicon Based Photonic Circuits," Nano Lett. **16**, 2289–2294 (2016).
[20] S. L. Mouradian, T. Schröder, C. B. Poitras, L. Li, J. Goldstein, E. H. Chen, M. Walsh, J. Cardenas, M. L. Markham, D. J. Twitchen, M. Lipson, and D. Englund, "Scalable Integration of Long-Lived Quantum Memories into a Photonic Circuit," Phys. Rev. X **5**, 031 009 (2015).
[21] E. Murray, D. J. P. Ellis, T. Meany, F. F. Floether, J. P. Lee, J. P. Griffiths, G. A. C. Jones, I. Farrer, D. A. Ritchie, A. J. Bennett, and A. J. Shields, "Quantum photonics hybrid integration platform," Applied Physics Letters **107**, 171 108 (2015).
[22] E. Bermúdez-Ureña, C. Gonzalez-Ballestero, M. Geiselmann, R. Marty, I. P. Radko, T. Holmgaard, Y. Alaverdyan, E. Moreno, F. J. García-Vidal, S. I. Bozhevolnyi, and R. Quidant, "Coupling of individual quantum emitters to channel plasmons," Nature Communications **6**, 7883 (2015).
[23] The identification of any commercial product or trade name is used to foster understanding. Such identification does not imply recommendation or endorsement or by the National Institute of Standards and Technology, nor does it imply that the materials or equipment identified are necessarily the best available for the purpose.
[24] K. Srinivasan and O. Painter, "Linear and nonlinear optical spectroscopy of a strongly coupled microdisk-quantum dot system," Nature **450**, 862–865 (2007).
[25] A. W. Elshaari, I. E. Zadeh, A. Fognini, M. E. Reimer, D. Dalacu, P. J. Poole, V. Zwiller, and K. D. Jöns, "On-Chip Single-Photon Sifter." Pre-print at https://arxiv.org/abs/1611.03245v3 (2016).
[26] K. Luke, A. Dutt, C. B. Poitras, and M. Lipson, "Overcoming Si_3N_4 film stress limitations for high quality factor ring resonators," Optics Express **21**, 22 829–22 833 (2013).

[27] H. Inoue, K. Hiruma, K. Ishida, T. Asai, and H. Matsumura, “Low loss GaAs optical waveguides,” IEEE Transactions on Electron Devices **32**, 2662–2668 (1985).
[28] J. Wang, A. Santamato, P. Jiang, D. Bonneau, E. Engin, J. W. Silverstone, M. Lermer, J. Beetz, M. Kamp, S. Höfling, M. G. Tanner, C. M. Natarajan, R. H. Hadfield, S. N. Dorenbos, V. Zwiller, J. L. O’Brien, and M. G. Thompson, “Gallium arsenide (GaAs) quantum photonic waveguide circuits,” Optics Communications **327**, 49–55 (2014).
[29] F. Marsili, V. B. Verma, J. A. Stern, S. Harrington, A. E. Lita, T. Gerrits, I. Vayshenker, B. Baek, M. D. Shaw, R. P. Mirin, and S. W. Nam, “Detecting single infrared photons with 93% system efficiency,” Nature Photonics **7**, 210–214 (2013).
[30] A. C. Dada, T. S. Santana, R. N. E. Malein, A. Koutroumanis, Y. Ma, J. M. Zajac, J. Y. Lim, J. D. Song, and B. D. Gerardot, “Indistinguishable single photons with flexible electronic triggering,” Optica **3**, 493–498 (2016).
[31] M. Köllner and J. Wolfrum, “How many photons are necessary for fluorescence-lifetime measurements?” Chemical Physics Letters **200**, 199–204 (1992).
